# Artificial Neural Networks: A Novel Approach to Analysing the Nutritional Ecology of a Blowfly Species, *Chrysomya megacephala*

**DOI:** 10.1673/031.010.5801

**Published:** 2010-06-09

**Authors:** André Bianconi, Cláudio J. Von Zuben, Adriane B. de S. Serapião, José S. Govone

**Affiliations:** ^1^ Departamento de Botânica, Instituto de Biociências — Unesp — São Paulo State University, Cep 13506-900, Avenida 24-A, 1515, Bela Vista, Rio Claro-SP, Brazil; ^2^ Departamento de Zoologia, IB, Unesp, Rio Claro-SP, Brazil; ^3^ Departamento de Estatística, MatemáticaAplicada e Computação, DEMAC, IGCE, Unesp, Rio Claro-SP, Brazil

**Keywords:** insect bionomics, larval density, life-history, mass rearing

## Abstract

Bionomic features of blowflies may be clarified and detailed by the deployment of appropriate modelling techniques such as artificial neural networks, which are mathematical tools widely applied to the resolution of complex biological problems. The principal aim of this work was to use three well-known neural networks, namely Multi-Layer Perceptron (MLP), Radial Basis Function (RBF), and Adaptive Neural Network-Based Fuzzy Inference System (ANFIS), to ascertain whether these tools would be able to outperform a classical statistical method (multiple linear regression) in the prediction of the number of resultant adults (survivors) of experimental populations of *Chrysomya megacephala* (F.) (Diptera: Calliphoridae), based on initial larval density (number of larvae), amount of available food, and duration of immature stages. The coefficient of determination (R^2^) derived from the RBF was the lowest in the testing subset in relation to the other neural networks, even though its R^2^ in the training subset exhibited virtually a maximum value. The ANFIS model permitted the achievement of the best testing performance. Hence this model was deemed to be more effective in relation to MLP and RBF for predicting the number of survivors. All three networks outperformed the multiple linear regression, indicating that neural models could be taken as feasible techniques for predicting bionomic variables concerning the nutritional dynamics of blowflies.

## Introduction

Being a mechanical vector of pathogenic microorganisms, *Chrysomya megacephala* (F.) (Diptera: Calliphoridae) is a blowfly of considerable medical and veterinary importance (Guimarães et al. 1978; Furlanetto et al. 1984; Laurence 1986; Lima et al. 1991; Gabre et al. 2005). *C. megacephala* is able to cause facultative myiasis in humans and animals, has an expanding geographic distribution, and was unintentionally introduced in Brazil in the 1970s (Zumpt 1965; Guimarães et al. 1983; Laurence 1986; Gabre et al. 2005). Moreover, the genus *Chrysomya* has been increasingly deployed in forensic studies for the determination of human postmortem intervals (Greenberg 1991; Catts and Goff 1992; Arnaldos et al. 2005; Gomes and Von Zuben 2005).

The larval phase of *C. megacephala* is deemed to be a critical developmental period in which intense limitation of resources frequently occurs (Levot et al. 1979; Goodbrod and Goff 1990; Reis et al. 1994). This limitation is conducive to dynamic competitive processes, wherein each larva attempts to feed off the available resources, scrambling to exploit the feeding substrate before the depletion of the food resource (Ullyett 1950; de Jong 1976; Lomnicki 1988; Von Zuben et al. 2001). Therefore, such exploitative conditions are embedded in interrelated processes that take place at the individual and population levels (Wijesundara 1957; Herzog et al. 1992; Von Zuben et al. 2001; Tammaru et al. 2004).

Regarding the nutritional ecology of immature blowflies, it has been suggested that the competition for food is influenced, concomitantly, by larval density and availability of food. Hence it is very useful and important to investigate the crowding level of immature individuals on the feeding resources by means of simultaneous variations of larval densities and amounts of food (Von Zuben et al. 2000; Ireland and Turner 2006).

The length of the larval stage of *C. megacephala* represents a useful variable for the adequate comprehension of its nutritional ecology as a whole. The importance of this parameter is strongly connected with the fact that individuals that require relatively long periods of larval development in order to reach their adult phases may undergo harsh feeding conditions (Von Zuben et al. 2001). Moreover, the accurate measurement of the larval phase duration may substantially contribute to the determination of the most cost-effective relationships between initial larval density and amount of available food in relation to feasible mass rearing techniques (Von Zuben et al. 2001; Tammaru et al. 2004).

The outcomes of exploitative competition for feeding resources determine the dynamics of population parameters such as survival, fecundity, weight, and size of the emergent adults (Von Zuben et al. 1993; Papandroulakis et al. 2000; Von Zuben et al. 2000; Ireland and Turner 2006). In relation to the resultant individuals, Von Zuben et al. (1993) regard the number of emerging adults as a variable that tends to decrease with an increase in the number of immature individuals of *C. megacephala.*

Under natural environmental conditions, there are several difficulties in conducting experiments and collecting suitable data on population bionomic features of *C. megacephala* (Gabre et al. 2005). Most natural situations do not allow researchers to obtain representative sample sizes or detailed descriptions of every developmental phase, whereas in laboratory conditions, immature specimens of *C. megacephala* feeding on a wide variety of substrates are able to develop into more advanced stages in an effective manner (Roy and Dasgupta 1971).

The complexities of the nutritional ecology of blowflies could be clarified and detailed by the deployment of appropriate modelling techniques such as artificial neural networks, which are mathematical tools widely applied to the resolution of complex biological problems. A notable feature of artificial neural networks is their independence from any assumptions about the theoretical distribution shape of the data used. Also, the performance of artificial neural networks over linear or non-linear regression-based statistical models is at least interesting, as the dimensionality and/or the non-linearity of the systems increase (Schultz and Wieland 1997; Haykin 1999; Schultz et al. 2000).

Neural networks algorithms are founded on the construction of models that consist of a great number of simple processing units called neurons (or nodes) that possess several connections between them and are lined up in layers. The number of neurons in the input layer corresponds to the variables that will be used to feed the neural network and should be the most relevant variables to the problem in question (Jang 1993; Haykin 1999).

Artificial neural networks were conceived with the aim of imitating the human brain functionality. Thus part of the terminology used in the area of artificial neural networks, namely neurons, synapses, learning, layers, etc., is due to such a fact.

However, it is important to emphasise that these terms are only associated with mathematical functions or the method of utilising them.

Regarding the use of neural networks in entomology, considerable improvements have been achieved in recent years. Obach et al. (2001) predicted the abundance of selected water insects in a small stream in central Germany by means of neural models. Using environmental data that were collected as part of a study of microhabitat use by butterflies, Bryant and Shreeve (2002) highlighted the potential for utilising neural networks in developing predictive models of microhabitat temperature. Several multidisciplinary studies have described the development of scale independent models, based around coupling artificial neural networks with climate-hydrological process models in order to simulate species' distribution, including insect species (Pearson et al. 2002; Pearson and Dawson 2003; Harrison et al. 2006). Worner and Gevrey (2006) used a self-organising map, which is an artificial neural network model, with the purpose of identifying global pest species assemblages and potential invasive insects, including dipteran species.

Furthermore, neural models were assessed in combination with a set of well-known ecostatistics in order to conduct function approximation (function fitting) and documentation based on invertebrate data (99 invertebrate families), including immature and adult specimens (Zhang and Barrion 2006). Howe et al. (2007) used neural networks to describe and predict insect body temperatures and insect behaviour in relation to environmental variables. Zhang and Zhang (2008) deployed neural models with the purpose of assessing the effectiveness of neural networks in modelling survival process and mortality distribution of a holometabolous insect (*Spodoptera litura*, tobacco cutworms) at different temperatures. Zhang et al. (2008b) utilised various neural networks in order to fit and recognise spatial distribution patterns of grassland insects. Nonetheless, apart from the present study, neural models have not been directly employed in the modelling of the nutritional ecology of blowflies.

Therefore, the principal aim of this work is to use well-known neural networks to ascertain whether these tools are able to outperform a classical statistical method (multiple linear regression) in the prediction of the number of resultant adults of experimental populations of *C. megacephala*, based on initial larval density (number of larvae), amount of available food, and duration of immature stages (in hours). Results obtained through statistical methods were compared with those derived from artificial neural networks algorithms for the purpose of achieving such a goal. Additionally, some basic concepts of neural models are outlined in order to permit entomologists to assess the potential of using artificial networks in the nutritional ecology of other insect species.

## Materials and Methods

### Formation of the laboratory generations

Adult individuals of both sexes were collected in Campinas (22° 49′ 9.52″ S and 47° 4′ 12.54″ W), São Paulo, Brazil. They were then identified and kept in nylon net cages (30 × 30 × 48 cm). Decaying organic matter, such as rodent and fish carrions, was utilised as bait. Prior to identification, the individuals were anaesthetised in a freezer at -18° C for 30 s. These insects were provided with water and refined sugar *ad libitum* and taken as the parental generation of this study. The cages were kept in a controlled temperature room (25 ± 1° C) at a relative humidity of 60 ± 10% and a photoperiod of 12:12 light:dark. In order to induce the development of the gonotrophic cycle, females were supplied with fresh macerated beef liver as a source of protein. For the formation of the next generations, ovipositions were obtained using small pots containing macerated decaying beef that were put into the cages in order to stimulate egg laying.

Different larval densities were formed using glass pots (8 cm in height × 7 cm in diameter) that contained four amounts (15, 30, 60, and 90 g) of an artificial diet (feeding resource) proposed and described by Leal et al. (1982). Such larval densities were based on F_2_ individuals (second laboratory generation), and five different proportions of larvae to amount of food were considered: 5, 10, 20, 30 and 40 larvae/g. Thus the larval densities utilised were: 75, 150, 300, 450 and 600 larvae, each in pots containing 15g of food; 150, 300, 600, 900 and 1200 larvae growing in pots that contained 30 g of food; 300, 600, 1200, 1800, and 2400 larvae in pots with 60 g of food; and 450, 900, 1800, 2700, and 3600 larvae in pots containing 90 g of the artificial diet.

The larvae counts were done using newly hatched individuals. Artificial diet pots were covered with organza and kept in a climatic room at 25 ± 0.2° C, 60 ± 10% relative humidity and 12:12 L:D. After the complete exhaustion of the food substrate, the pots were put into bigger plastic pots (20 cm in height × 12 cm in diameter) with a 5 cm-thick sawdust layer (pupation substrate). Organza was removed from the small pots in order to enable the larvae to move through these pots toward the sawdust. The bigger plastic pots were covered with organza and kept at identical experimental conditions.

### Variables

In this study, the number of survivors (resultant adults) was the dependent variable (output), and the others were considered the independent or explanatory variables (inputs), namely larval density (initial number of larvae), amount of available food (in grams), and duration of immature stages (from the first instar larval stage to the final pupal stage, in hours). Such variables were primarily chosen based on their biological significance for the comprehension of the nutritional ecology of blowflies. Combinations of the three input variables provided 40 values (sample size) concerning the output variable.

**Figure I.**
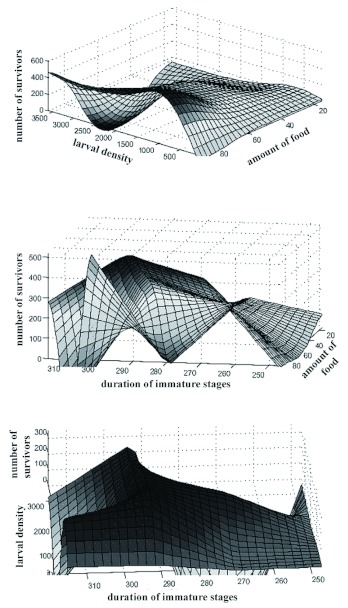
Representation of the number of survivors (actual values) as a function of *Chrysomya megacephala* larval density (initial number of larvae), amount of food (in grams), and duration of immature stages (from the first instar larval stage to the final pupal stage, in hours). High quality figures are available online.

The whole data set (*n* = 40) provided a coefficient of determination of 0.59 (*F* = 30.94, p < 0.001) prior to splitting the original data set into smaller subsets by means of neural network techniques. A stepwise linear regression (forward selection) revealed that the joint contribution of larval density and duration of immature stages to the linear regression was equal to 2%. This low value, albeit statistically significant (p < 0.001), did not represent the practical importance of such variables because it is biologically inconceivable that the number of resultant adults could vary only as a function of the available amount of food, irrespective of variations in larval density and duration of immature stages (Levot et al. 1979; Getz 1984; Von Zuben et al. 1993, 2000; Ireland and Turner 2006).

**Figure 2.**
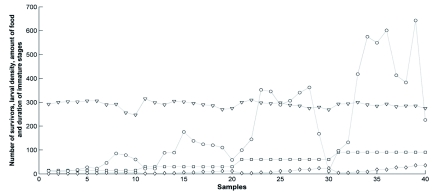
Representation of the whole data set (*n* = 40) prior to neural network procedures. Samples (x-axis): each combination of input values is shown. The y-axis shows the number of survivors (dependent variable) and the three input variables (independent variables). 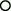 is the number of survivors (output variable), 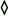 is the larval density (initial number of larvae). 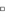 is the amount of food (in grams). 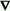 is the duration of *Chrysomya megacephala* immature stages (from the first instar larval stage to the final pupal stage, in hours). Values concerning the larval density were divided by 100 for ease of presentation. High quality figures are available online.

Each independent variable (input) produced distinct output responses (Figure 1). On the whole, such explanatory attributes generated non-linear variation in the number of survivors. Additionally, an overall lack of strong linear relationships between input and output variables was observed (Figure 2), and the accuracy of conventional regression methods may have been significantly reduced in the presence of non-linearity (Neter et al. 1996; Schultz and Wieland 1997). Moreover, the p-values concerning the significance of the input variables were based on underlying statistical assumptions (Neter et al. 1996). On the other hand, artificial neural networks are able to model complex nonlinear systems, even when the exact nature of any relationships is unknown (Schultz and Wieland 1997; Schultz et al. 2000; Bryant and Shreeve 2002; Howe et al. 2007; Zhang et al. 2008b). From a biological point of view, all the explanatory variables are considerably important, and neural models permitted the utilisation of such variables, irrespective of their statistical significance.

### Basic concepts of the utilised neural network models

Neural networks technology utilises a multilayered approach to approximating complex mathematical functions in order to process data. It consists of many processing elements (nodes or neurons) that work in a parallel manner. Neurons are connected to each other in layers that are interconnected. The connections between neurons weight the data transformation process of each neuron, sending the information to the next node or output layer. Such connections are known as synaptic strengths or weights. The training process starts by furnishing the neural network with a variety of examples (called training sets). The data sets normally contain input and output data. The neural model creates connections and is able to learn patterns based on the relationship between input and output data sets via the adaptation of the synaptic weights to changing inputs.

Three well-known neural networks, namely Multi-Layer Perceptron (MLP) (Haykin 1999), Radial Basis Function (RBF) (Wasserman 1993), and Adaptive Neural Network-Based Fuzzy Inference System (ANFIS) (Jang 1993), were used to compare their outcomes with those derived from the deployment of multiple linear regression (a classical statistical method). The coefficient of determination (R^2^), which lies within the interval 0 ≤ R^2^ ≤1, was utilised for comparing such outcomes. A zero R^2^ indicates that the predictive model does not explain the variance of the actual data set.

**Figure 3.**
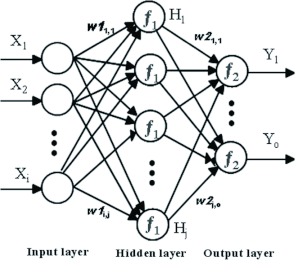
A typical three-layer feed-forward Multi-Layer Perceptron network architecture with *i, j*, and *o* neurons in the input, hidden, and output layers respectively, *f_i_*, represents the activation function, *w* stands for the weights. *Xi* represents the input variables. *Yi* stands for the output variables. High quality figures are available online.

The statistical assumptions of the multiple linear regression model and its feasibility followed the detailed descriptions in Neter et al. (1996). A basic description of the neural models will be made in the following sections.

### Multi-Layer Perceptron (MLP)

MLP is the most widely employed type of feed-forward neural network (Haykin 1999). MLP networks consist of an input layer, one or more hidden layers, and an output layer (Figure 3).

Each layer has a number of processing units, and each of them is fully interconnected with units in the subsequent layer by means of weighted connections in a “cascade” manner, without any connections between neurons in the same layer. The number of neurons in the input layer (*X*) is equal to the number of variables of the problem in question. Also, there is an output layer (*Y*) in which the network response is made available and the number of neurons is equal to the desired number of quantities derived from the inputs. With respect to a regression-based analysis, there is a single neuron in the output layer. The layers between input and output layers are called hidden layers (*H*).

The computation of final output values is conducted in a layer-by-layer manner. Accordingly, each neuron of a specific layer other than the input layer computes a linear combination of outputs of the previous layer. Secondly, the resultant values are multiplied by the weight of the connection (*w*). Finally, such products arrive at each hidden neuron and are summed. Furthermore, there is a possibility of incorporating a shift called bias (*b*) into the neuron inputs. Each neuron calculates its output value by means of the corresponding activation function (*f*). Then, those values are continuously propagated toward the next layer, thereby reaching the output layer. Activation and output functions that are used frequently encompass linear (identity) functions, sigmoidal (S-shaped) functions, such as the logistic function, and the Heaviside thresholding function.

The training process is conducted as follows (Haykin 1999): a pattern is presented to the inputs. This pattern is transformed during its passage through the layers of the network toward the output layer. Then, the outputs of the network, as they are in this phase, are compared with the outputs that ideally would have been encountered if this pattern had been exactly stated. Based upon such comparisons, all the connection weights (*w*) are modified to some degree to ensure that the same pattern could be presented to the inputs. The differences (errors) between the actual outputs and the desired outputs are propagated backward from the top layer to lower layers in order to modify the connection weights. Generally, steepest descent techniques would achieve a suitable performance if local minima were relatively distant. On the other hand, they require a lot of iterations to converge when minima are near. A number of different types of back-propagation learning algorithms have been proposed, such as the Levenberg-Marquardt (Hagan and Menhaj 1994), with the aim of finding an optimum solution to a minimisation problem. It utilises an approximation to the Hessian matrix updating Newton-like weight.

### Radial Basis Function (RBF)

**Figure 4.**
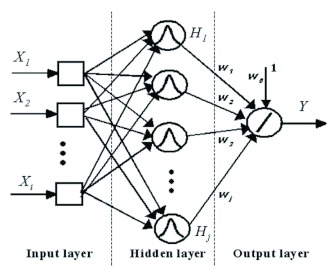
A typical Radial Basis Function network architecture with *i* and *j* neurons in the input (*X*) and hidden (*H*) layers, respectively, and a single neuron in the output layer (*Y*). An activation function (drawn inside the circles) depicts what happens at a given neuron. *Wis* represent the weights. High quality figures are available online.

RBF networks were introduced into the literature in the late 1980s (Broomhead and Lowe 1988; Poggio and Girosi 1990). Such neural models are non-linear hybrid networks that represent an approach to universal function approximation, and they were first used to solve multivariate interpolation problems (Poggio 1994). Furthermore, they are able to approximate a wide class of non-linear multidimensional functions. These neural networks are deemed to be a special class of multilayer feed-forward networks. They consist of a fully connected architecture with an input layer, a hidden layer, and an output layer (Figure 4).

There is one neuron in the input layer for each predictor variable. The input neurons (X_i_) act as an input data buffer and do not execute any processing. The hidden layer has a variable number of neurons that should be determined by the training process. The neurons in the hidden layer contain the gaussian function as an activation function, and their outputs are inversely proportional to the distance from the centre of the neuron. Each neuron consists of an RBF centred on a specific point that possesses a number of dimensions that should be equivalent to the quantity of predictor variables. The spread (radius) of the RBF function may be different for each dimension. When this network is presented with the *X* vector of input values derived from the input layer, a hidden neuron computes the Euclidean distance from the test case to the central point of the neurons and then applies the RBF kernel function to this distance, using the spread (σ). The resultant value is transferred to the output layer. A hidden neuron is more sensitive to data points near its centre. Regarding the gaussian RBF, such neuron sensitivity may be tuned by adjusting the spread, whereby larger spreads indicate less sensitivity. In the output layer, the neurons implement a weighted sum of hidden unit outputs (linear combination of hidden functions).

During the training process, the parameters of the RBF are the number of neurons in the hidden layer, the coordinates of the centre of each hidden-layer RBF function, the radius (spread) of every RBF in each dimension, and the weights that were applied to the RBF function outputs in the summation layer.

There are several training algorithms for the RBF networks. In contrast to other networks, the training process is divided into two phases: (1) determination of the parameters of the basis functions, and (2) computation of the output weights. During the first phase, clustering methods for finding central positions of the radial basis function can be used with a specific number of hidden units. Regarding fixed basis functions in the second phase, the weights have been traditionally optimised by the least mean square algorithm or by other optimisation methods. The number of hidden layers is dynamically adapted in response to the output error. Further information concerning RBF learning algorithms may be found in Schwenker et al. (2001).

### Adaptive-Network-based Fuzzy Inference System (ANFIS)

A fuzzy inference system is a framework that entails fuzzy logic, fuzzy decision rules, and fuzzy reasoning (Takagi and Sugeno 1985). It consists of a left-half side (also called “if” or “antecedent” side) and a right-half side (also called “then” or “consequence” side). Conventionally, each linguistic state variable has one or more fuzzy sets that are represented by a linguistic “value.” Such fuzzy sets are characterised by associated membership functions over the Universe of Discourse of a specific variable. A state membership value (µ(X)) represents the “degree of membership” of the state variable *x* in a fuzzy set (linguistic value), or the “degree of truth” of *x*, taken as an actual value. The outcome is a number within the interval [0,1], in which 1.0 signifies a full membership. Each rule may include every combination of state (µ(x)) and input memberships (µ(u)) on the left-half side and must incorporate a state membership value calculation (µ(x)) into the right-half side, indicating how an alteration in a specified state could occur.

**Figure 5.**
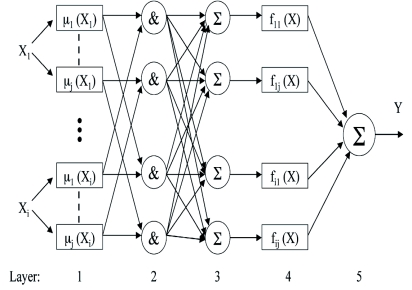
A typical schematic representation of an Adaptive-Network-Based Fuzzy Inference System. 1 : “values” layer. µ(Xi): membership values. 2: “rules” layer. 3: normalisation layer. 4: function layer. 5: output layer. High quality figures are available online.

An ANFIS is a first-order Sugeno-type fuzzy inference system where the membership function parameters are fitted with a specific data set by a hybrid-learning algorithm (Jang 1993). Its structure (Figure 5) consists of a first layer (values layer), in which the nodes represent sets of each state variable (input). At this same layer, membership values are computed, and the membership functions are deemed to be adaptable. The second layer (rules layer) implements the t-norms for modelling the logical fuzzy “AND” operator and computing the rule matching factor. The third layer (normalisation layer) acts to scale the firing strengths (matching factors).

The output of the fourth layer (function layer) is comprised of a linear combination of the inputs multiplied by the normalised firing strength *w*:

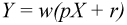
where *p* and *r* are adaptable parameters. The fifth layer (output layer) is a simple summation of the outputs of the fourth layer. The adjustment to modifying parameters is a two-step process. First, information is propagated forward through the network to the fourth layer, where the parameters are identified by a least-square estimator. Then the parameters in the second layer are modified using gradient descent backpropagation. The number of membership functions in the Universe of Discourse for each input and the output training information must be specified at this phase.

### Experimental procedures for analysing the data set

All the tests and results were derived from programming Matlab 6.5®. An optimum network and parameter configuration for each of the three networks deployed was established by trial and error. The input layer of every network utilised in this study consisted of three input nodes, representing the independent variables (initial number of larvae, amount of food, and duration of immature stages). The output of the network models was the dependent variable (number of survivors).

It has been suggested that a one-hiddenlayer MLP permits the approximation of any continuous function, provided that an adequate number of nodes in this layer are found; two hidden layers are sufficient to furnish a “best” approximation for any nonlinear mapping (Cybenko 1989; Haykin 1999). The introduction of additional hidden layers in the network architecture could allow the resolution of more complex problems. Nevertheless, such an introduction reduces the generalisation ability of the network, while the training time increases (Foody 1995). Haykin (1999) stated that the number of hidden nodes should be as small as possible while still allowing the network to retain a performance close to the optimum. Because the prediction of the number of survivors (output) may be adequately represented by a continuous function (Figure 2), the MLP network was deployed with one hidden layer.

The number of nodes in the hidden layer of the MLP network and the stopping criteria were optimised with the purpose of obtaining precise and accurate output values. The hidden layer consisted of five neurons. The activation function of the hidden layer was the hyperbolic tangent sigmoid function, and the linear function was used for the output neuron. The Levenberg-Marquardt training algorithm (Hagan and Menhaj 1994) was selected. During the training processes, the stopping criteria fixed the number of epochs at 2000. The target error for this phase was set at 0.001.

The number of hidden nodes of the RBF network was automatically found to be 25. The outputs of the hidden layer neurons were determined by the Euclidean distance between the network input and the centre of the basis function. The spread of the gaussian function was empirically set at 0.78. The RBF network output was formed by the weighted sum of the hidden layer neuron outputs and the unity bias. Additionally, the target error deployed was the same used with the MLP network.

Regarding the ANFIS model, three bellshaped membership functions (low, medium, and high) were determined for the number of larvae and amount of available food, whereas four triangular membership functions (very short, short, long, and very long) were set for the duration of immature stages. The optimisation method used in the training of the input membership function parameters was the Backpropagation learning algorithm. The output membership functions were linear zeroth and first-order Sugeno-type system. The number of epochs was fixed at 500, and 0.001 was taken as the target error; the other parameters were determined by trial and error in order to reach the best performance. The membership functions were learned from an adaptive neuro-fuzzy inference system. Furthermore, a set of 36 fuzzy rules was implemented, and their weights were adjusted in order to feasibly model the training data.

The entire data set was split into two subsets that were used for training and testing each neural network. Accordingly, the whole data set (*n* = 40) was divided randomly into two subsets, namely the training and testing subsets. Specifically, the ratio of training examples to testing examples was 30:10. Regarding the multiple linear regression, the training and testing subsets were established identically. The ratio of 30:10 was deployed in each training procedure with respect to all the models. Furthermore, a cross-validation strategy was not utilised, and the estimation errors could vary depending on the data subset.

The inputs and targets were normalised in order to have zero means and unity standard deviations. Moreover, the outputs were trained to produce outputs with zero means and unity standard deviations. Each network converged after reaching the maximum number of epochs. The training error was 0.0129 for MLP and 0.0409 for RBF. Then the network outputs were restored to their original values (raw data) in order to calculate the R^2^ between estimated and observed values of the training and testing subsets.

## Results

Table 1 shows the coefficients of determination (R^2^) and root-mean-square errors that were obtained from the data subsets utilised both in training and in testing procedures in every model, including the multiple linear regression. As a whole, the linear regression exhibited the lowest accuracy (the lowest R^2^ and highest root-mean-square error values).

Regarding the neural networks, the R^2^ derived from the **RBF** (0.715) was the lowest in the testing subset, even though its R^2^ in the training subset exhibited virtually a maximum value (0.999). All the models had lower R^2^ values (less accuracy) in the testing subset compared with the training subset.

**Figure 6.**
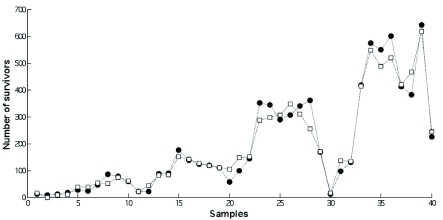
Actual and predicted number of survivors (output). The actual values were derived from the original data set (*n* = 40), and the predicted values were obtained from the Adaptive-Network-Based Fuzzy Inference System network (the most accurate model), 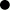 represents the original data set (raw data), and 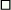 represents the predicted values. Samples: each combination of input values is shown. High quality figures are available online.

The ANFIS model permitted the achievement of the best testing performance (the highest R^2^ and the lowest root-mean-square error). Hence, this model was deemed to be more effective than the MLP and **RBF** networks in predicting the number of survivors. The output values predicted by the ANFIS model fitted the actual data (original values) in a satisfactory way (Figure 6).

## Discussion

The sample size utilised in the present work (*n* = 40) may be sufficient to conduct most ecological and biological approaches concerning the nutritional ecology of blowflies. Nevertheless it is important to note that larval density and amount of food (two of the input variables) provided the multiple regression model with few distinct values (Figure 2), because only 11 densities (75, 150, 300, 450, 600, 900, 1200, 1800, 2400, 2700 and 3600 larvae) and four distinct amounts of available food were utilised (15, 30, 60 and 90g), making up the five proportions of larvae per gram of food used in this work (5, 10, 20, 30 and 40). Therefore, the larger amount of distinct values measured for the output variable (resultant adults), using the same combination of values of those input variables, caused a lack of fit that decreased the prediction capability of the models.

Nonetheless, the amount of distinct values of larval densities deployed in the present study was considerably larger than those widely used in experimental designs on nutritional ecology of blowflies (Mackerras 1933; Slansky and Rodriguez 1987; Goodbrod and Goff 1990; Von Zuben et al. 2000). Furthermore, owing to operational limitations, such studies would be impracticable if numerous levels of larval density were used (Von Zuben et al. 1993, 2001). Thus, the use of artificial neural networks would be justified in this case, since the neural algorithms coped with the lack of fit better than the multiple regression method did (Table 1).

The RBF network exhibited a less accurate performance (lowest R^2^ and highest rootmean-square error in the testing subset) in the present study, considering only the three neural network models that were employed. On the other hand, in a study conducted by Zhang and Barrion (2006) based on invertebrate data sampled in an irrigated rice field, RBF was considered an effective approach to function approximation and documentation of sampling information. Such results may serve as a basis for further discussions about the feasibility of RBF networks in entomology in terms of their underlying attributes and robustness.

Howe et al. (2007) modelled the body temperature and activity of a widespread butterfly species (*Polyommatus icarus*) in relation to weather, with the aim of predicting how future climate may influence its activity. These authors utilised a multilayer feed-forward backpropagation network in order to accomplish their objectives, and this neural model was deemed to be superior to a generalised linear modelling approach to predicting body temperature. In the context of nutritional ecology, the MLP network performed better than two other models, namely, the linear regression and the RBF network (Table 1).

Zhang et al. (2008b) investigated the spatial distribution pattern of grassland insects by means of neural models. They concluded from their results that neural networks were more flexible than a conventional model. Additionally, these authors indicated that further research based on more complex distribution patterns should be conducted with the aim of obtaining more reliable conclusions. Similarly, neural networks were deemed to be superior to a conventional model (linear regression) in the current study. Nevertheless, the present work utilised simple experimental designs and only three explanatory variables (input). Therefore, more complex neural network studies should be implemented in order to explain the portion of the total variance that was not accounted for by the models. Furthermore, larger sample sizes are highly desirable because the number of examples (sample size) that is usually utilised in nutritional ecology may prevent the full utilisation of the potential of neural networks.

Owing to the relatively small number of distinct values, the input variables of larval density and amount of food did not permit the “best” possible fit, including with the neural models, between predicted and collected values. Nonetheless, their incorporation into the modelling was very important. If duration of immature stages were the only input variable, the modelling would have considerably lost its biological meaning (Getz 1984; Arditi and Saïah 1992; Papandroulakis et al. 2000; Von Zuben et al. 2000). Moreover, it would not have been possible to establish the suitable larval density and amount of food that could determine a particular number of resultant adults (Von Zuben et al. 2000).

**Table 1.**
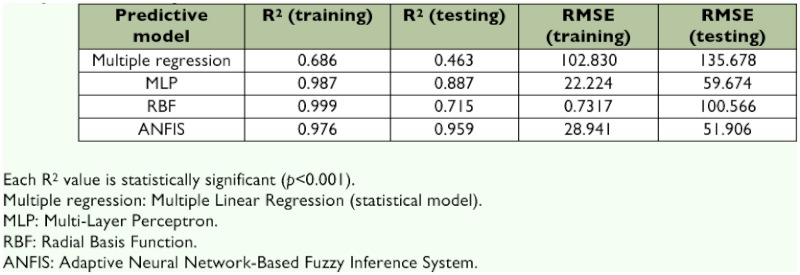
Coefficients of determination (R^2^) and root-mean-square errors (RMSE) for each model deployed both in the training and in the testing subsets.

The ANFIS network exhibited the most accurate performance in the testing subset, although further studies may be necessary for corroborating this superiority in other practical experiments. On the whole, the neural networks outperformed the multiple linear regression (Table 1), indicating that neural models could be taken as feasible techniques for predicting bionomic variables concerning the nutritional ecology of blowflies. The present study constitutes only a first approach, albeit with promising applicability, in which the ecology of blowflies was effectively analysed by means of neural models.

The employment of a simple linear regression requires a nontrivial amount of statistical expertise. The use of a multiple non-linear regression model such as an MLP requires more knowledge and experience (Sarle 1994). Therefore, it is important to highlight the usefulness of multidisciplinary studies with the aim of conducting effective investigations of bionomic parameters.

Future studies may consider other complex variables that were not assessed in the current work. Ambient temperature, for example, was utilised by Zhang et al. (2008a). These authors deployed neural network algorithms (functional link artificial neural network) in order to model the food intake dynamics of larvae of *S. litura* (Lepidoptera). Six different temperatures were used for measuring the food intake, and the neural network approach was deemed to be accurate.

It is possible that unresolved problems concerning the bionomics of immature and adult individuals of blowflies could be disentangled and clarified by the use of neural models. The employment of such complex analytical tools may help entomologists to feasibly schematise the sort of practical situations in which the utilisation of artificial networks is able to provide new insights into the nutritional ecology of blowflies.

## Abbreviations

ANFIS: Adaptive Neural Network-Based Fuzzy Inference System;
MLP: Multi-Layer Perceptron;
RBF: Radial Basis Function
